# Airport route development strategy planning and performance measurement with a dynamic performance management framework

**DOI:** 10.1371/journal.pone.0271452

**Published:** 2022-07-12

**Authors:** Jie Feng, Cheng-Lung Wu, Jinfu Zhu

**Affiliations:** 1 College of Civil Aviation, Nanjing University of Aeronautics and Astronautics, Nanjing, China; 2 School of Aviation, UNSW Sydney, Kensington, New South Wales, Australia; DePaul University, UNITED STATES

## Abstract

Route development programs have become popular with airports worldwide to enhance air connectivity. Enhanced air connectivity of an airport has been found to have a positive and significant impact on the competitiveness and attractiveness of the airport and the regional economy in which the airport is located. However, the drivers behind route development programs and their performance are not always clear; this has been less explored in the literature. In this paper, a dynamic performance management approach is taken to build a modeling framework in which key drivers affecting route development performance and strategic resources affecting key drivers are identified. Route-level planning is used in an empirical study to demonstrate the dynamic mechanism of airport route development initiatives and performance measurements. The proposed framework can not only provide performance monitoring, but also suggest suitable indicators to evaluate the performance for policymakers in future airport route development.

## Introduction

Air service development and/or route development (RD) is a well-known practice in the airport industry [1–3]. Originally, “air service development is a broad term that encompasses a variety of activities with the ultimate goal of retaining existing air service or improving air access and capacity in order to develop the economy of a community or region” by the definition of the National Academies of Sciences, Engineering and Medicine [4]. More recently, the Copenhagen Economic Report stated, “Route development activities are those marketing activities undertaken by airports with the aim of attracting new routes, for example through participation in route development conferences, offering incentive schemes, meetings with airlines, producing bespoke reports for airlines etc.” [5]. Concurrently, “air service incentives are financial inducements offered to airlines to encourage new services to particular airports and to mitigate some of the financial risks that an airline takes when it starts a service in a market that it did not previously serve” [6].

Nowadays, RD is considered an essential tool of airport marketing strategy for airlines aiming to build new connections or further develop existing routes [1, 7–9]. This refined marketing strategy is usually directed by airports or other non-government organizations, given the significant positive impacts of air services on airport activities, economies, business, and communities [6]. Predominantly, it has been frequently used by larger airports. This is possibly because airport managers expect that the incentivized or cooperated airlines will bring more traffic, which might generate large commercial revenues to make them self-sufficient. The success of Singapore Airport has made evident the decisive role of its flag carrier, particularly in developing an integrated and complex network, which helps to gain competitive advantage [10, 11].

Airport network connectivity has been viewed as an important factor driving airports’ attractiveness to travelers and competitiveness in the industry [7, 12–15]. Theoretically, an accessible and interconnected network will enhance airports’ attractiveness; the more attractive the airport, the better its competitive position relative to other surrounding airports serving the same region. The competitive position helps an airport to earn market share and improves productivity through higher passenger volume and freight volume. The increased traffic can leverage commercial revenue for larger airports, but has a lower contribution to regional airports. However, well-connected airports can enhance the national air network efficiency and the development of communities and businesses. Hence, enhancing air connectivity via RD programs has been considered a common strategy for modern airports.

Several studies have examined the measurement of air connectivity (see, for example [7, 16–18]). Although past studies have focused on various aspects of airport RD—such as mathematical models, measurements, and applications—the impacts of RD on airport connectivity have not been explicitly considered. No study has formally modeled the potential airport attractiveness and the subsequent productive and financial performance brought by airport connectivity enhancement. The existing literature on route impacts has mostly focused on national networks and hub fostering (notably, see [18]). Therefore, these studies did not directly examine the effects and mechanisms through which RD influences airport connectivity and the performance of an airport business.

Allroggen et al. [19] investigated factors affecting the introduction of RD and indicated that the main driving force for the presence of incentives for route and traffic development is the characteristics of an airport, which also influence the objectives of an RD program [20]. Empirical studies and surveys have been conducted to study the broad application of airport incentive programs in the European Union (EU) and the United States (US) [1, 3, 6, 21], and the importance of cooperation among stakeholders toward successful RD [22–24]. Malina et al. [3] highlighted the importance of RD impact measurement. However, few studies have formally evaluated the effects of RD. Ryerson [21], the National Academies of Sciences, Engineering and Medicine [6], and Feng et al. [25] evaluated the impacts of RD.

RD is a complicated process, and some airports have to use multiple incentives to entice airline cooperation [1, 20, 26]. Studies under the airport–airline arrangements context have shown the positive impacts of incentives on airport–airline collusion [27–30]. Such studies, however, cannot fully explain the increasing use of incentives for RD in the airport industry and the systematic process of RD, including the formulation, implementation, and performance estimation. This is possibly because RD programs are often more specific to the business environment of individual airports. Complementary studies are needed to provide the theoretical support for RD by developing models that incorporate the resources and efforts required to plan RD airport businesses and communities.

This paper aims to explain how route resources can make airports more connected, attractive to consumers, productive, commercially viable, and beneficial to communities. This requires all stakeholders (such as airports, governments, airlines, and local communities) to think beyond their governance boundaries and to perceive the value of RD in the airport ecosystem context, in which air services often represent “livelihoods services” and/or significantly contribute to airport profitability, regional economic, and social development [31, 32]. In particular, this paper emphasizes the system processes regarding resources alignment within an airport business and stakeholders’ efforts, leading to the improved productivity and financial performance of an airport business owing to enhanced airport connectivity and attractiveness to customers. The dynamic performance management (DPM) approach is employed to model the RD processes and simultaneously consider all stakeholders and their influences for program planning and evaluation. An empirical study is conducted to demonstrate the application of the DPM-based models in RD planning and performance measurement.

The present study aims to make both methodological and policy contributions to the literature. First, to the best of our knowledge, this paper is the first to explore value creation and transformation between an airport business and its users in the RD context. Such value analysis contributes to theoretical explanations for RD, which is not well grounded from the perspective of a system process. Second, DPM approach-based models have been built to support practitioners in planning and evaluating RD. We believe that we have designed innovative tools to systemically explore RD from the outcome-based performance perspective, which was not adequately addressed in the literature. Third, we use an empirical study on a Chinese airport to demonstrate various aspects of policy development in airport RD planning. Since each airport has a unique business environment, this case study used the DPM approach to highlight government regulations on airport charging, RD resources, and the role airlines could play in a successful RD program. The proposed DPM model can help provide insights for improving government regulations on airport charging reforms to provide more flexibility for establishing airport RD programs under complex business environments. Moreover, this case also geographically broadens prior empirical studies in the literature, which have largely focused on the US or EU airports.

The structure of this paper is as follows. Section 2 explains the DPM method used for the following exploration. Section 3 outlines the value transformation during an RD program, which lays the foundation to build generic models in Section 4 in terms of different perspectives, ranging from airport ecosystem levels to RD programs. Section 5 is an empirical study that uses the proposed DPM model to conduct RD planning for new international destinations. Section 6 summarizes the results and presents the concluding remarks.

## Research method: Dynamic performance management framework

The DPM approach, a performance management (PM) method combining system dynamics (SD) modeling, was recently developed by Bianchi [33]. A PM system is crucial to planning competitive strategies and measuring outcomes through identifying the results and their own drivers [33]. Traditional PM cannot capture the dynamic complexity of managerial decisions; this usually results in performance traps and paradoxes [34]. The SD model developed by Forrester [35] is a modeling technique for complex dynamic systems and could be applied to consider the interactions between agents and dynamic effects over time. Particularly, the feedback analysis of SD modeling allows decision-makers to better allocate strategic resources and frame strategic structures according to the performance behavior of the dynamic system [36]. Thus, the combination of PM and SD modeling will help address issues arising from the traditional PM, and support decision-makers to better understand the cause-and-effect relations among strategic resources, performance drivers, and end results [36, 37].

**Fig 1:**
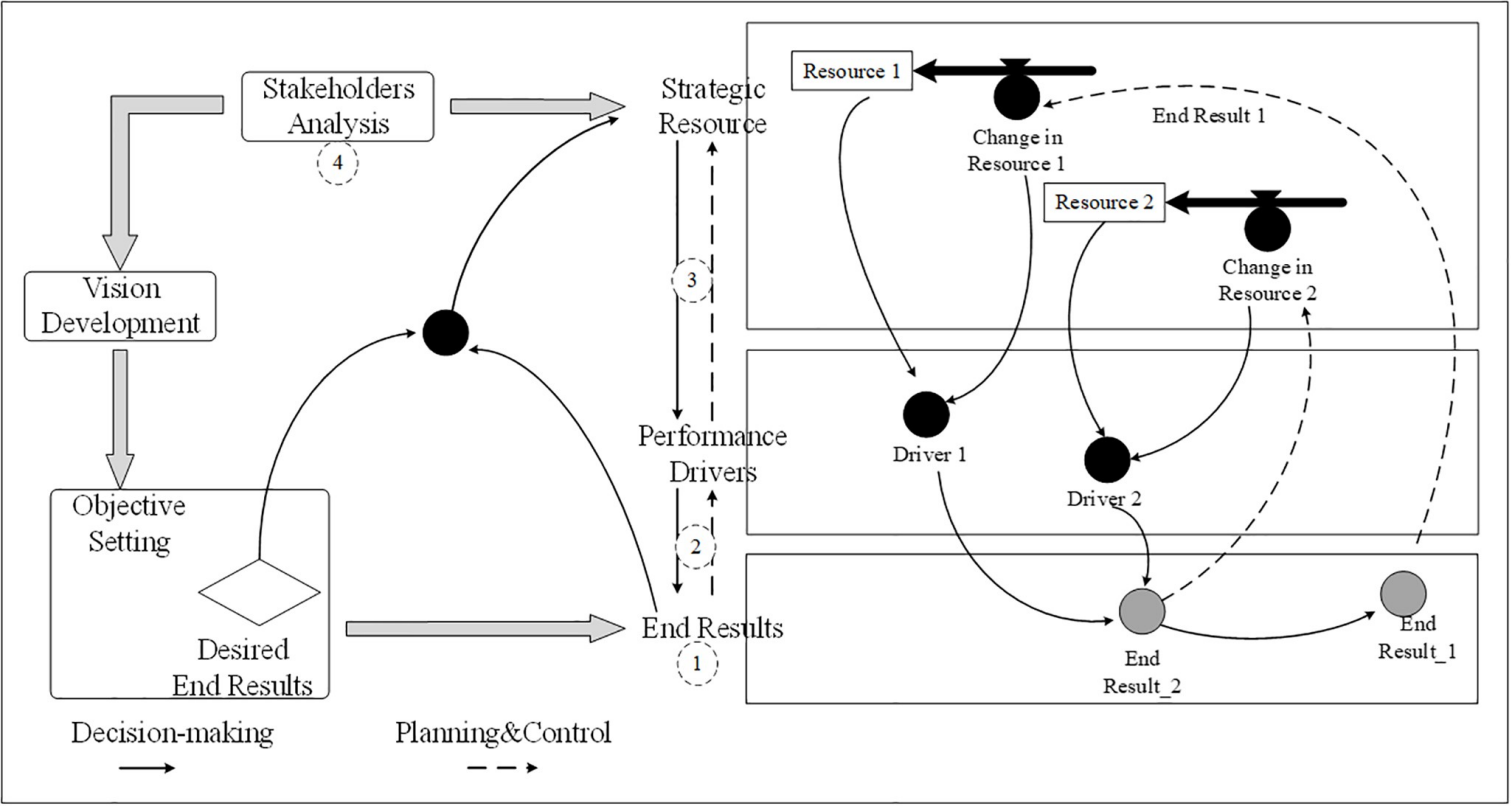


There are three main variables in the DPM model framework: strategic resources, performance drivers, and end results. Strategic resources refer to physical or information resources held by the system and can be deployed to generate desired results [34]. In practice, resources are not independent and they can affect one another [33]. In terms of performance drivers, they represent the performance indicators that benchmark the intermediate results and end results. These are the outcomes or results that the organization/system obtains through the management cycle. Thus, by the identification of end results, related performance drivers, and strategic resources, a DPM model can be built (as shown in Fig 1).

The accumulation and depletion of strategic resources will be affected by the endogenous impacts of the end results. The value of resources in a given time is determined by the strategic policy developed by decision-makers. Then, the resources can be deployed to enhance performance drivers and the intermediate results. Ultimately, this affects the end results. Consequently, organizations will achieve sustainable development via the positive interaction cycles among resources, performance drivers, and end results [33].

DPM emphasizes the achievement of the end results and tracks backward to define the performance drivers (or intermediate results) and their connections to the deployment of strategic resources. Hence, the “end results → performance drivers → strategic resources” logical structure will help decision-makers identify the driving forces and their dynamic mechanism toward achieving the expected performance. DPM has been used in public utilities and enterprises [36]. In particular, Noto [34] first used DPM in urban transportation planning and demonstrated the advantage of DPM in supporting decision-makers at both managerial and political levels from a dynamic and systemic perspective. Notably, Bivona and Cosenz [38] explored multiple platform business models by using the DPM approach to disclose the explicit relationships between strategic resources, the value creation and capture drivers, and the way such drivers influence platform performances. Their study demonstrated the application of the DPM framework in the investigation and management of the cause-and-effect mechanisms. This not only clarified the dynamic value creation and transformation among resource and performance drivers, but also identified critical drivers to the success or fail of multiplatform business.

Air transport is a complex system involving various stakeholders as well as the airport system [39]. Because of the changing operational environment, diverse stakeholders and conflicting objectives and interests in policy-making are common issues faced by managers and practitioners in the airport industry. Therefore, introducing a systematic dynamic thinking and modeling approach in the context of the airport system is required. Although using SD is not new [39–41], the use of DPM-based models in airport PM is yet to be explored. Moreover, airports have been proved a two-sided platform, on which airlines and passengers join to interact [42]. The use of DPM in the airport system will help us understand the characteristics of different airport business models as a two-sided platform. Further, it will also frame and assess strategic effects from the perspective of outcomes, and identify the outcome-oriented drivers for airport businesses.

## Value creation and transfer under route development

RD is generally used as a marketing strategy by airports to airlines aiming to build new connections or further develop existing routes [1, 8, 9]. Airport connectivity change is the apparent result, particularly for regional airports; airports in practice anticipate the transformation by the connectivity enhancement toward airport productivity and financial performance improvement [43]. Increased traffic brought by incentivized airlines can stimulate the growth of non-aeronautical revenue, which has a greater contribution to the long-term financial sustainability of an airport business. For example, the RD implemented by Narita International Airport in Japan successfully increased routes and flights. In this context, Narita initiated the marketing incentives with the objective to promote retail sources and other non-aeronautical enterprises (available on the official website). The incentives for airlines in this case were paid on the basis of the “number of departing passengers handled” and “increase in passenger numbers” separately for international and domestic routes.

**Fig 2:**
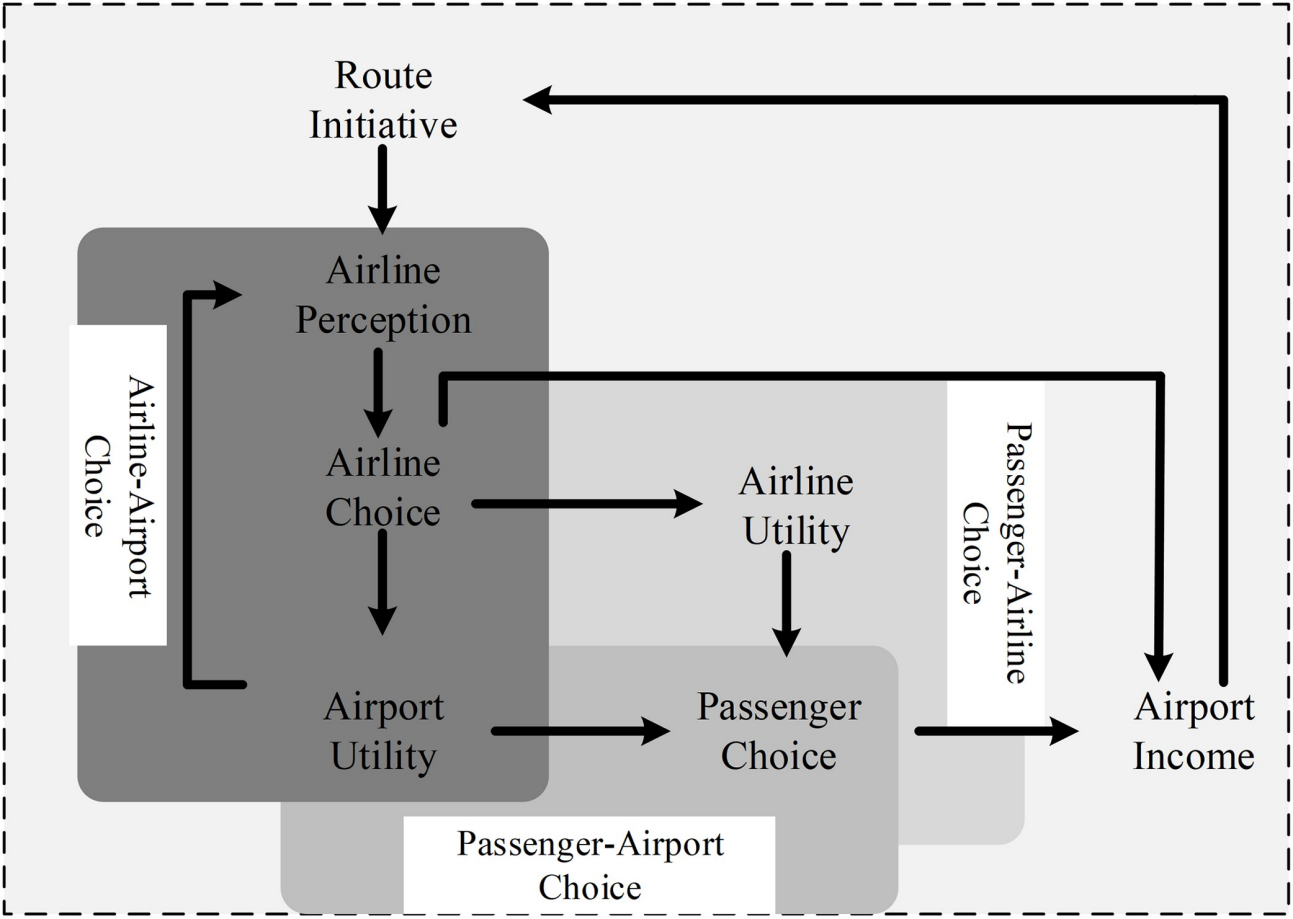


In such an environment, aeronautical customers (airlines) and non-aeronautical consumers (passengers) both contribute to enhance and co-create service and product values for airports. Thus, airports have been considered and validated as a two-sided platform instead of a vertical structure [42, 44]. Apart from obtaining revenue from airlines and passengers, the role of airports is to facilitate airlines to provide passengers with air services, and foster a profitable interaction among them, leading to passengers’ satisfaction. Particularly, under RD initiatives, airlines are also the partners who contribute to enrich airport services, which attracts more airlines and reinforces airport competition and network effects. In this value chain, passengers are no longer the “passive” consumers located at the end of the value chain because their experience also adds value to airports’ service and products. Therefore, on the basis of the <<resources–drivers–outcomes>> logic corresponding to the <<value creation–value capture–outcomes>> process, the value creation, capture, and transfer mechanism of airport RD is illustrated in Fig 2.

The framework in Fig 2 identifies all participants and connections of the values created and captured among them. With the intent to improve financial sustainability, airports invest in RD strategies via incentives, by which the value can be created to drive airline decisions. The prime cause of this action is that airports’ charging accounts for a large portion of airlines costs. Hence the RD incentives, mainly in the way of airport service charging discount, can be considered as risk-sharing for airlines’ new services preference. This, in turn, is shown in enhanced connectivity to improve the attractiveness of an airport to influence passengers’ airport choice decisions because new air services will promote airport accessibility. Meanwhile, new services connected by airlines also improve their utilities to affect passengers’ airline choice, particularly when airlines pass on financial benefits received from airports to passengers by lowering airfares. Eventually, together with the incentive associated with airport connectivity, routes affect traffic, which, in turn, influences financial viability for stakeholders, which may endow RD with more funds. Therefore, the increased route value captured by airlines and airports can further attract and crowd the corresponding market separately via incentives to new services.

RD is a complex process. This value-chain mapping emphasizes that the resources derived by end results can create value, which, in turn, can be captured as the outcome to affect the end results. The implementation of RD requires airports to make explicit the value creation provided by all stakeholders and the transfer and exchange of captured values inside the airport performance system. Hence, a sustainable and profitable business cycle can be developed, as shown in Fig 2.

## Dynamic performance management approach to model airport route development program

To use the DPM decision-making approach to explain the dynamic interactions of airport RD programs, some key elements need to be identified, including outcomes, performance drivers, resources, and stakeholders. Using the value framework provided in Fig 2, we first identify the end result of the airport RD program as the attractiveness of an airport to airlines and passengers. A conceptual model is developed to explore drivers behind airport attractiveness, and then used to identify the required resources and stakeholders involved in the airport system. Then, a DPM-based model is built to describe the dynamics in the airport system, focusing on the effects of an airport RD program.

### DPM key factor identification for route development

**Fig 3:**
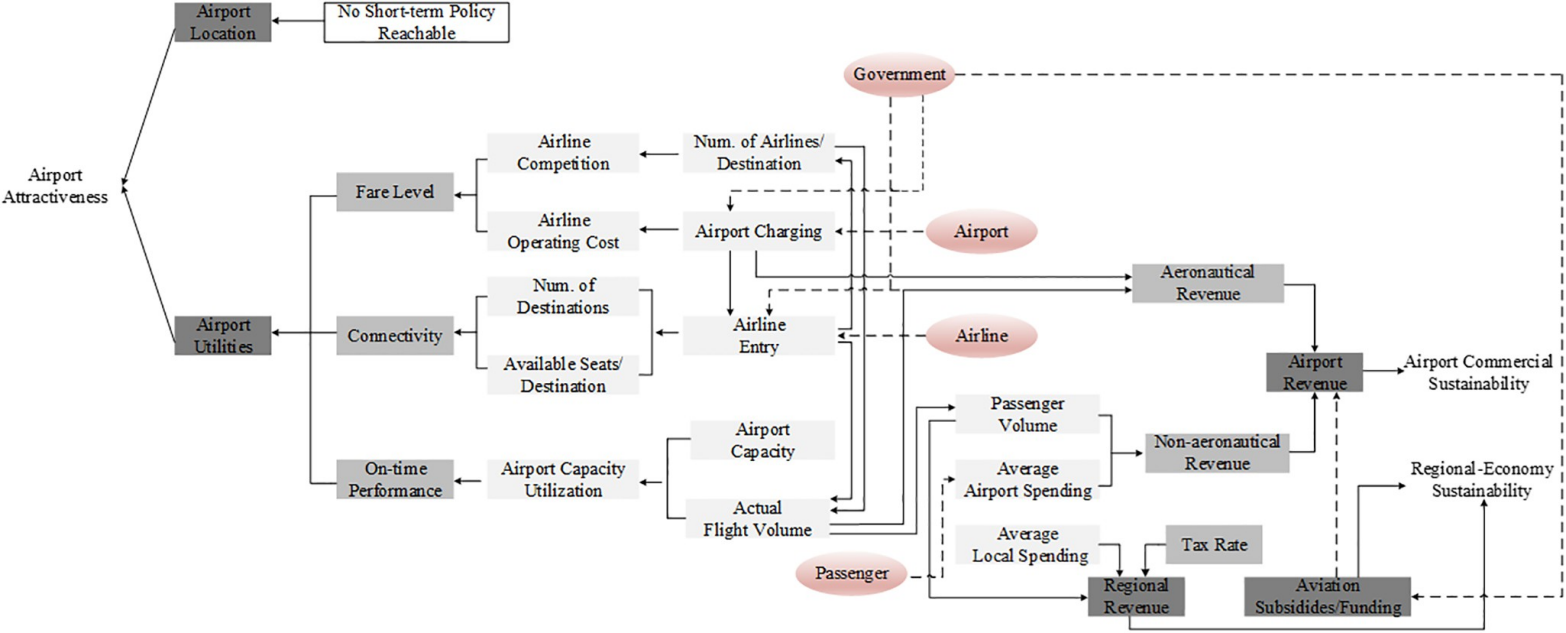


Driving factors influencing airport attractiveness are identified and used to track resources and stakeholders. While airport attractiveness is generally conceptualized as driven by straightforward and unambiguous criteria[14, 17, 45], from the perspective of passenger choices, it could be influenced by the location of an airport, and other utilities passengers gain from services of a particular airport (as illustrated in Fig 3). Location and related factors such as ground transport services are often beyond the control of airports. Hence, airports tend to focus on strategies that can enhance other airport utilities, such as fare level, connectivity, and on-time performance by resident airlines [14].

Factors determining connectivity play a key role in describing airport utility, as shown in Fig 3. The number of airlines and serving flights of a particular airport creates competition among airlines, which affects fare level, the number of routes/flights, airport capacity utilization, and consequently the on-time performance of flights. Meanwhile, flights served by airlines improve airport attractiveness and airport financial performance by increased passenger volume and the subsequent increase in airport revenues. The more connected an airport’s network is, the more attractive it is relative to other competing airports in the same region. In addition, the improved network may enhance service level by the airport and stimulate economic growth of the region with more air traffic flow. Thus, routes could be considered a crucial resource and revenue generator for airports. Hence, the conceptual model in Fig 3 can explain why RD is the focus of interest to airport managers and authorities, and provide insights for building the DPM model focusing on the airport RD program. Moreover, we can identify main stakeholders (in pink bubbles) who have the power to manage and allocate various resources.

As noted above, airport attractiveness is determined mainly by three factors, and they are not independent. Within the airport system, air connectivity has various definitions and calculation methods [7, 16–18, 46]. In general, air connectivity is determined by the number of connections and number of flights, which are directly provided by airlines at an airport to passengers. Airport-fare level is mostly the decision of airlines, which determine the ticket price with consideration to operating costs and market competition. Airport fees account for a large proportion of airline operating costs and affect airfare levels. In terms of on-time performance, airport operating efficiency has effects on on-time performance. In particular, airport capacity utilization affects flight on-time performance and is determined by how the capacity is used by scheduled and operating fight volumes. Therefore, RD influences these three factors directly, which consequently affect airport attractiveness.

### DPM-based generic models

**Fig 4:**
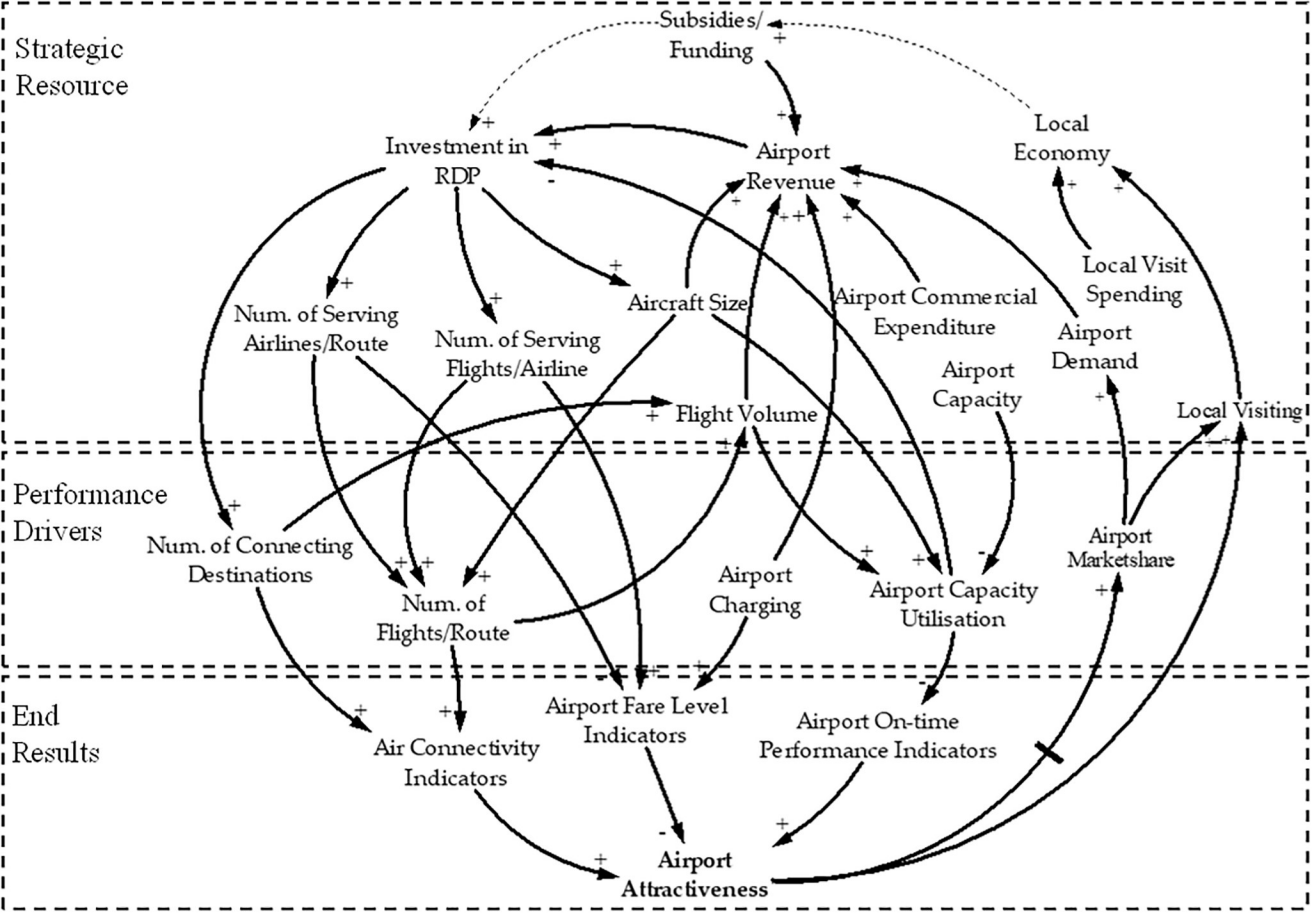


Within the framework of attractiveness criteria above, Fig 4 illustrates a generic DPM-based model of RD programs by airports or local governments to improve airport attractiveness on the airport system level. The main objective of RD is to enhance airport attractiveness (the end result), which is influenced by three intermediate results: air connectivity, airport-fare level, and on-time performance from the bottom-up (as observed in Fig 4). The three intermediate results are influenced by four performance drivers: the number of connected destinations, the number of flights/routes, airport charges, and airport capacity utilization. Those performance drivers are affected by the allocation of resources from the top level, which provides the airport with revenues that are generated from airport demand. Meanwhile, airport demand is stimulated by the enhanced attractiveness and will generate more airport revenue, which can be used for RD program investments to reallocate resources to enhance performance drivers. Hence, the dynamic process will generate a virtuous cycle to maintain the profitable sustainability of airports.

Fig 4 indicates that two main resources should be considered within the airport system: financial resources and capacity resource. Financial resources will determine the nature of RD, which is either airport-directed or government-directed (in dotted line in Fig 4), because the money used for route investment can be constrained under various legislations, regulations, and objectives. For the government-directed funding source, this highlights the contribution of air services to enhance local economic development by leveraging increased traffic for local airports where airports are usually underutilized. The airport-directed funding source helps change airport productivity and profit sustainability; airports that use this type of scheme are often large airports that might be facing capacity constraint and may not be subsidized by government funding. Nevertheless, the reciprocal and cooperative relationship between local economic growth and airport development has been evidenced theoretically and empirically [47]. Hence, from the perspective of airports, government subsidies used to develop routes could be considered an exogenous policy assistance (in dotted line) to help airports achieve self-sufficiency within the airport ecosystem [31].

Fig 4 depicts the resources needed to conduct RD, the possible outcomes, and end results that can be used to systematically evaluate the effectiveness of RD on the system level. These analyses and process-mapping graphs support the detailed RD program planning, including objective setting, marketing investigation, activities undertaking, and program implementation [8]. Fig 4 shows that factors affecting air connectivity drivers have divergent effects on the other two performance drivers—fare level and on-time performance. Hence, policy designed to change air connectivity will ultimately change airport attractiveness via internal relationships. Therefore, on the program level, comprehensive strategies should aim to enhance the connectivity drivers that stimulate end results. Air connectivity is typically influenced by the number of destinations, the number of airlines per route, the number of scheduled flights, and the aircraft size deployed by airlines. These drivers also explain the various objectives of existing RD programs—to change the number of connections, flights, or passengers [3].

**Fig 5:**
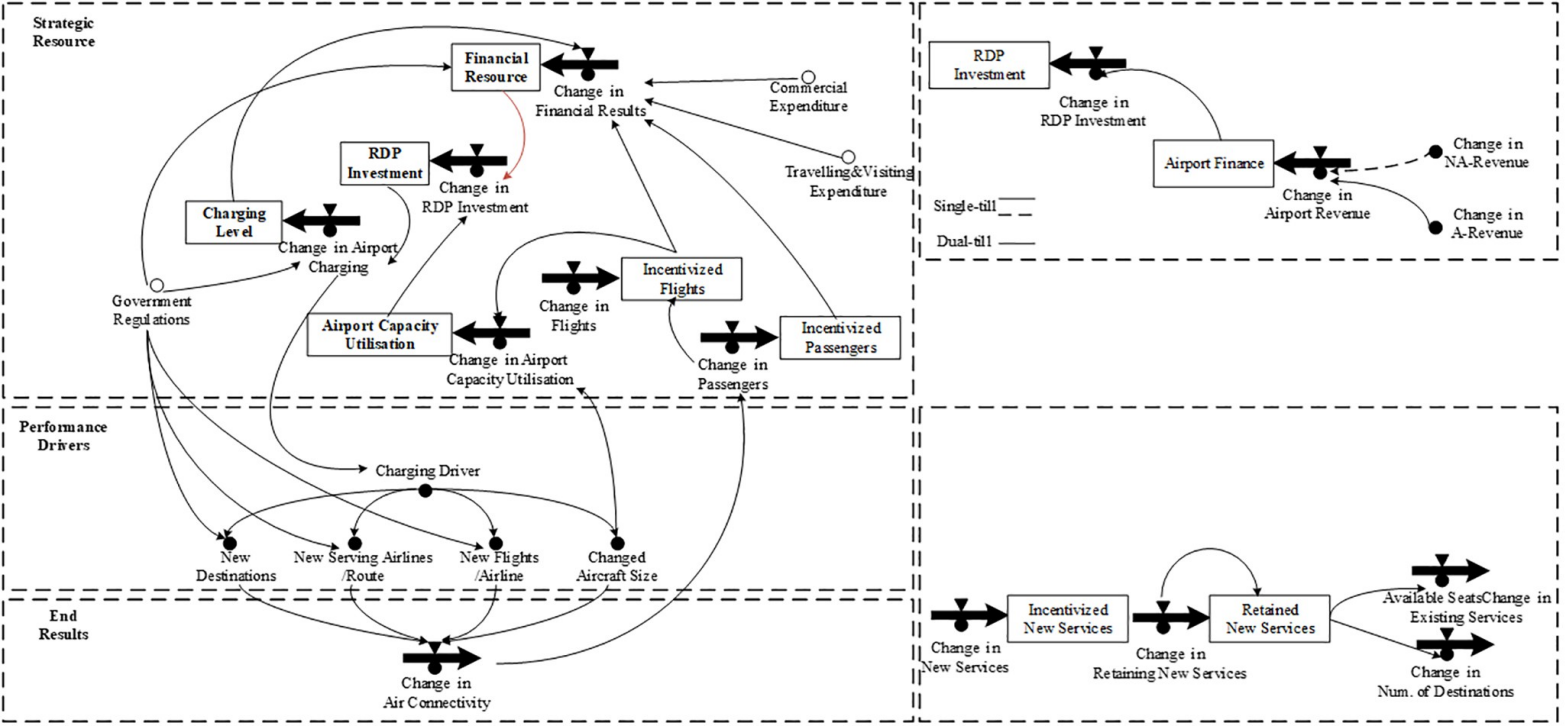


RD planning on the program level is outlined in Fig 5, which illustrates the end results, performance drivers, and strategic resources on the basis of the DPM modeling approach. Although air connectivity is the end result for most airports, airports might have different operating situations and various business objectives. Some airports, large and profit-oriented in particular, prefer high-yield traffic by attracting international air services and services to business-oriented markets [6, 20]. Other airports might aim to increase traffic volume via attracting low-cost carriers to maximize runway utilization and airport terminal facility utilization. All performance drivers are determined by airline services, which are charged service fees by airports. Charges paid by airlines become airports’ revenue to cover costs and influence airlines’ operating costs to make service planning decisions (performance drivers), especially when airlines adopt the cost-efficient business model. Therefore, charging drivers are the crucial factor influencing other performance drivers, as shown in Fig 5, such that airports can negotiate charging fees with airlines. This cause–effect relationship demonstrates and explains charging discount tactics used by airport RD programs.

Although most airports now operate under a more relaxed regulatory environment, government regulation still plays a vital role, especially when airports’ decisions are financial. Financial resources used for RD not only define the characteristics of RD, as discussed above, but also raise concerns regarding airport charging regulations, as shown in the top-right section of Fig 5. When an airport uses the single-till charge framework, revenue earned from non-aeronautical revenue could be used as investment in RD incentives; this is usually done in the form of aeronautical fee waivers for airlines. Hence, cross-subsidies should be evaluated to explore whether the revenue sourced from the aeronautical part of business could be compensated by non-aeronautical incomes.

Another concern is the effectiveness of RD programs. The retained air services during or after an RD program will ultimately influence connectivity and generate long-term effectiveness for route and traffic development [19]. However, it is often difficult to identify and estimate those long-term effects because the growth of traffic and new connections can be attributed to many other economic and political factors. Hence, on the program level, indicators shown in the bottom-right section of Fig 5 could be used to estimate the effectiveness of RD programs for future planning and peer benchmarking purposes. Specifically, these indicators could also help airports identify airlines that could potentially take advantage of incentives because those airlines usually stop services once the incentives terminate.

The DPM framework has been applied in this study to explore and visualize the dynamic process of RD on the airport system and program levels. The DPM-based models allow managers and practitioners to plan and evaluate RD programs using a systemic approach to identify resources and stakeholders and to detect and predict possible results via performance metrics. Using the DPM modeling approach, we were able to model all the important agents/factors within the system to analyze their roles in RD programs. Therefore, the generic models under different levels will not only help decision-makers understand the complexity of airport systems, but also provide a learning tool to design and implement RD programs. When specific quantitative data are not available for analysis in this framework, qualitative modeling is the first step for practitioners and managers to learn the trade-offs between resources and stakeholders by collecting and analyzing feedbacks. This qualitative approach also provides a conceptual model ready for more sophisticated simulations in the future for policy improvement and measurement, such as by system-dynamic simulations [48].

## Case study: Hangzhou Xiao Shan international airport

**Fig 6:**
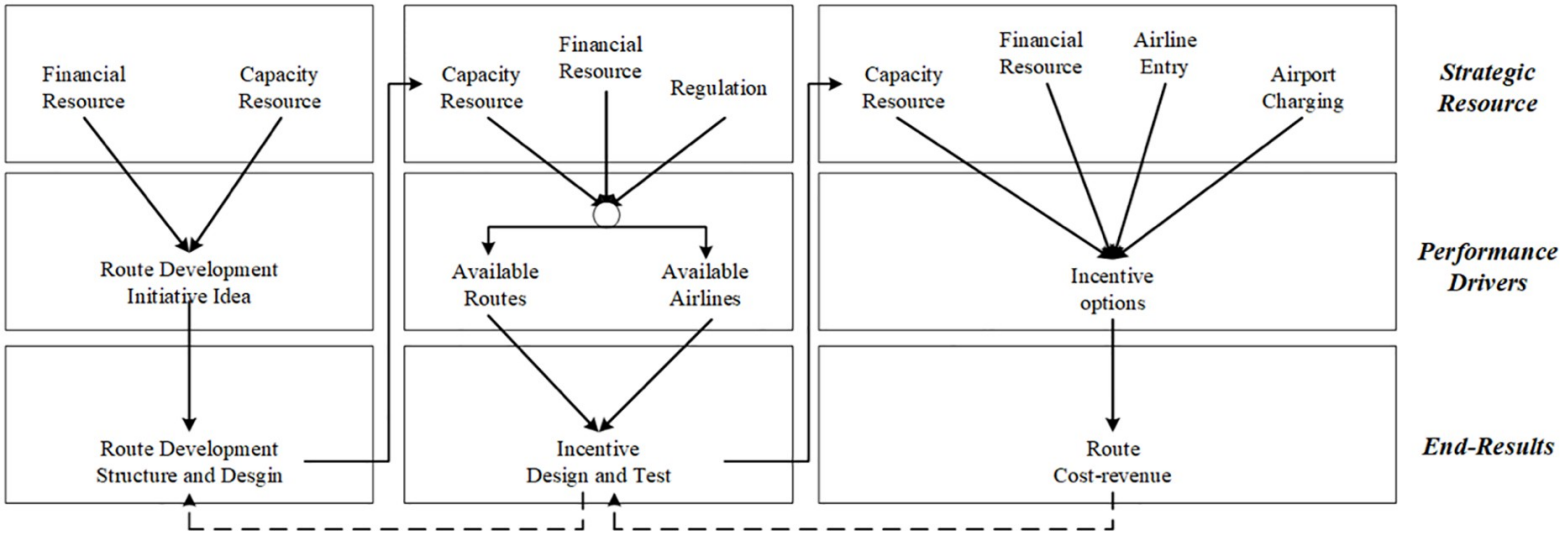


In this section, a case study will illustrate how those models could be used for an airport to plan RD programs. On the basis of the theory of DPM, resources should be first analyzed to estimate the possible outcomes of performance drivers and the end results. Fig 6 shows how the RD strategy planned from the airport level to the program level and then to the lower route level will generate feedbacks to identify end results and corresponding strategic resources. Hangzhou Xiao Shan International Airport (HGH) in China has been selected for empirical analysis. However, owing to limited accessibility to financial data, we will conduct the analysis on the program level and the exploration and calculation on the route level.

### HGH airport background

**Fig 7:**
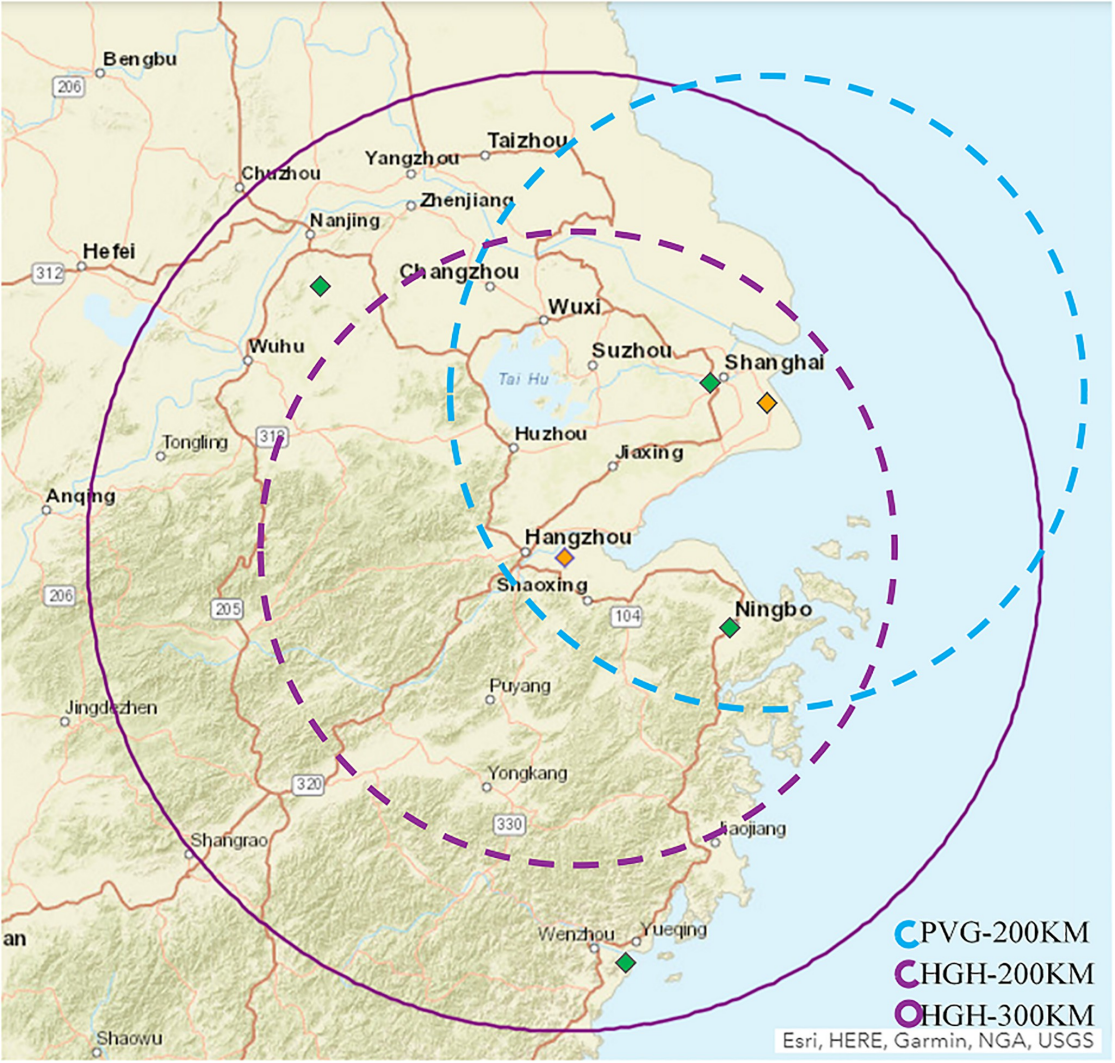


HGH is the busiest airport of Zhejiang Province, in the Yangtze River Delta Region. This region has been proposed as one of the mega regions by the Chinese central government and is considered one of the largest metropolitan areas in the world. The developed economy in this region has stimulated strong travel demand such that the multi-airport and advanced high-speed rail network have provided multi-modal choices for flexible movements of the population [49, 50]. However, the high-speed rail network also causes intense competition between airports serving the same hinterland. Within this region, HGH is facing competition not only from international hubs and regional hubs such as Shanghai airports (Pudong International Airport (PVG) and Hongqiao International Airport) and Nanjing Lukou International Airport, but also from internal provincial airports such as Ningbo International Airport and Wenzhou International Airport. The area served by HGH and the relative distance of surrounding airports are shown in Fig 7, which suggests that HGH and PVG have fierce competition because they are located within a 200 km catchment area.

Although HGH has ranked among the top 10 airports in China and has evident increases in passenger, flight, and cargo volumes in the past years, the growth of new connecting cities, especially international cities, has been slow. The official website of HGH shows that it has built connections with 62 international cities, including most short-haul services for the Asia region but less intranational long-haul services to the US, EU, Oceania, and Africa. The tourism data from Zhejiang Province show that this province has the highest market demand for international travel, particular for the EU and US markets. The connectivity gap of HGH, particularly for the international market, has resulted in leakage of potential passengers to nearby airports, such as PVG, which has more international flights. Simultaneously, the less connected international network at HGH could motivate interested stakeholders to take action to enhance airport attractiveness via building air connectivity. Therefore, financial and capacity resources can be estimated and deployed so that those changes could affect new routes, which will ultimately make HGH more attractive.

### RD planning for HGH

According to the system and program level models proposed above, airport capacity and financial resources used for RD, and government resources, such as entry and charging regulation, should be analyzed before the development of the detailed strategy plan. In regard to the financial state of HGH, the HGH bond credit rating report (S1 in S1 File) asserted that HGH earned a profit during the 2016–2019 period and had no unrecovered losses; the prominent core business has continued to make profits. Within the core business, non-aeronautical business income increased remarkably compared with aeronautical business income. In addition, positioned as the regional hub by the Civil Aviation Administration of China (CAAC) and considered the economic engine of Zhejiang Province, both the national and local documents have provided favorable policy environments for HGH. In particular, Zhejiang Province provided the funding to support the construction of transportation infrastructures—the reconstruction and expansion project of HGH—to promote capacity, which will provide more runway and terminal resources to satisfy future development.

In terms of government regulation, charging rules and route regulation are the most related polices, which are under separate official documents. According to the renewed airport charging document published by the CAAC in 2017 (S2 in S1 File), local airports could lower and raise aeronautical charges for domestic flights on the basis of the prescribed level but routinely could only lower them for international flights. This pricing framework grants more price flexibility and permits local airports to better exploit their market power. In terms of the route resource regulation (S3 in S1 File) renewed recently, the approval rules prefer to permit new international routes launched in the regional hubs and secondary airports to optimize the national network and achieve maximum utilization of airport resources. Further, the CAAC regulated that “carrier serving new routes will be permitted to enjoy market monopoly for three years.” This rule provides airports an advantageous condition to negotiate with airlines to build networks; the monopoly operation will reduce the financial risk of airlines when establishing new connections.

After the resource and regulation environment, and HGH’s network analysis, the details of the RD strategy should be planned on the basis of the expected performance to enhance connectivity. Particularly, financial support should be determined considering airport cost–revenue and airlines’ willingness to service because the purpose of RD is to “align both airports’ strategic development aims and airlines’ scheduling and network planning decisions” [3]. The duration, market specificity incentive mechanisms, and types of services and growth [3] will be identified by the RD budget and used to draft eligible rules for incentives. Incentives could be provided in various forms, such as discounts on certain fees and bonus payments, and the discount amounts could be calculated according to HGH’s operating data [1, 3].

In terms of RD duration, there is no clear consistency in the research literature. Governments and airports worldwide have various schemes. For example, Narita International Airport in Tokyo, Japan, has launched two 3-year RD programs on the official website (S4 in S1 File). Airports in the EU provide incentives for different durations, ranging from 1 year to 5 years [3, 26], while FAA’s document has regulated that the duration of air service incentives directed by airports in the US should be no more than 2 years. In China, many local province governments have provided airlines with a 3-year subsidy to foster international long-haul markets (S5 in S1 File). The CAAC provides a 3-year monopoly market operating environment for new international routes. Time durations on government documents should be thoroughly considered according to the local aviation industry.

The above analysis suggests that HGH should focus on the international market, especially the long-haul international market, because of the service gap between the high demand for international long-haul travel to the EU and US and fewer connecting flights. This suggestion is well supported by the operation data from Variflight, which show that 94.5% of flights in HGH were served by A320 and B737 during the first 8 months in 2018 [51], owing to the high percentage of domestic destinations and short-haul international destinations connected by HGH. Therefore, we assume that HGH will first focus on building new connections to expand international long-haul networks and emphasize the growth of passenger volume. In terms of incentive types, we assume that HGH will provide discounts on landing and taking-off fees or passenger service fees, which is under the regulation of the CAAC. We assume that HGH will provide a 3-year incentive for airlines (international and domestic) to launch new international services. All the assumptions for the incentives are shown in Table 1, which will be used to complete the new route scenario analysis.

**Table 1:** Assumptions for international route development analysis in the HGH case study.

**Operating airlines**	**Domestic**	√	--
	**Foreign**	--	√
**Service type**	**Pax**	√	√
	**cargo**	--	--
**Market specific**	**Domestic**	--	--
	**International**	√	√
**Growth type**	**Network**	√	√
	**Volume**	--	--
**Incentive mechanism (Discount item)**	**Landing and taking-off fee**	√	√
	**Passenger service fee**	√	√
**Duration of incentives**	**Three years**	√	√

### HGH new route selection, assumptions, and dataset

Using the high-demand traveling data from Zhejiang Province, we selected Paris (Paris–Charles de Gaulle [CDG]) as the route planning empirical study to show the cost–profit of new routes. This new route will be served by one domestic airline and one foreign airline on the basis of the air service agreement between China and France, which contracts that direct flights will be served by one Chinese airline and one French airline. In terms of charging rules, international flights operated by domestic and foreign airlines have the same level of fees, involving landing and taking-off fees (hereafter landing fees), parking fees, bridge fees, and passenger service fees (PSF) and security charges (PSC). During flight operations, landing and taking-off fees are charged per movement by the current regulation. While passenger service fee will be charged twice because both arriving and departing passengers will use the facilities within the airports. The detailed charging items are shown in Table 2.

**Table 2:** Charging process in HGH.

Charging Process	Landing & Taking-off Charge (LTC)	Parking Charge (PC)	Boarding Bridge Charge (BBC)	Passenger Service Fee (PSF)	Passenger Security Charge (PSC) ^!!^
Process					
**Arrival**	√^!^	√	√	√	
**Departure**				√	√

^**!**^ The charge is calculated according to the MTOW in the certificate of airworthiness expressed in ton and payable for each movement.

^!!^Airport security charge includes Passenger and Baggage Security Charge and Cargo and Mail Security Charge, but only passenger related security charge has been considered in the calculation.

CAAC has published the charging rules for flights operated by foreign and domestic airlines (S2 Table in S1 File). We assume that A330–200 with 264 seats deployment will be used for both airlines. Further, an industry standard seat load factors (SLF) of 70% was selected, resulting in 184 passengers loads. To ensure comparability, aircraft type, operating period, flight frequency, air bridges, and parking time will be constant and consistently applied to the new route’s flights schedule. Average passenger cost and commercial revenue (CR) will be calculated separately by dividing annual total aeronautical costs and non-aeronautical revenue related to passengers by annual passenger volume. The basic charging price is shown in Table 3 (all the monetary values calculated in this work are based on the currency USD/CNY = 6.7518, which was published by Tourzj in 2017 to show the foreigner average travel expenditure.).

**Table 3:** Basic charging fee for one movement.

Route: CDG-HGH-CDG			
Charging items	Standard (USD)	Charging items	Standard (USD)
***Runway Charges***		***Pax Charges***	
**Landing fee**	$1588.91	**Pax service**	$3815.28
		**Pax security**	$327.02
***Terminal Charges***			
**Airbridge**	$88.87		
**Aircraft parking**	$238.34		
**Total airport charges**	$1916.11	Total passenger charges	$4142.30
**Assumptions**:			
**Aircraft**: A330-200 **MTOW**: 238.0*T* **Available Seats**: 264.0			
**Seat Load Factor**:70.0% **Passengers**: 184.0			
**Parking time**: 3.00 Hours ^$^ **Bridge time**: 3.0 Hours			
**Average operating cost**: $9.47 **Average commercial revenue**: $2.38^$$$^			
**Average travel expenditure**: $683.64			

^$^ The time for parking and air bridge have been set as three hours, according to the maximum connecting for international service.

^$$^ This value is published by Tourzj on the official website.

^$$$^ Non-aeronautical revenue is the sum of renting and VIP lounge income based on the income items of HGH.

Operational and financial data published on the official website of Hangzhou Xiao Shan International Airport, and the credit rating report published on the official website of United Credit Rating Co. Ltd., will be used. Data from other relevant municipal government documents and annual statistical reports have been collected and reviewed. The tourism data from the Zhejiang Province Tourism (Tourzj) have been used to analyze traveling trends and identify the new route. This also provides the data source to calculate and estimate the contribution of inbound passengers to local economic revenue. In addition, the regulation documents published by the CAAC—including the airport charging rules and certificate of airworthiness—provide references from which to calculate airport revenues and passenger traffic. Data from 2017 are used to conduct the following analyses owing to the limited consistency and accessibility of HGH financial data.

### Scenarios analysis for new route planning

#### Analyses for new routes

**Fig 8:**
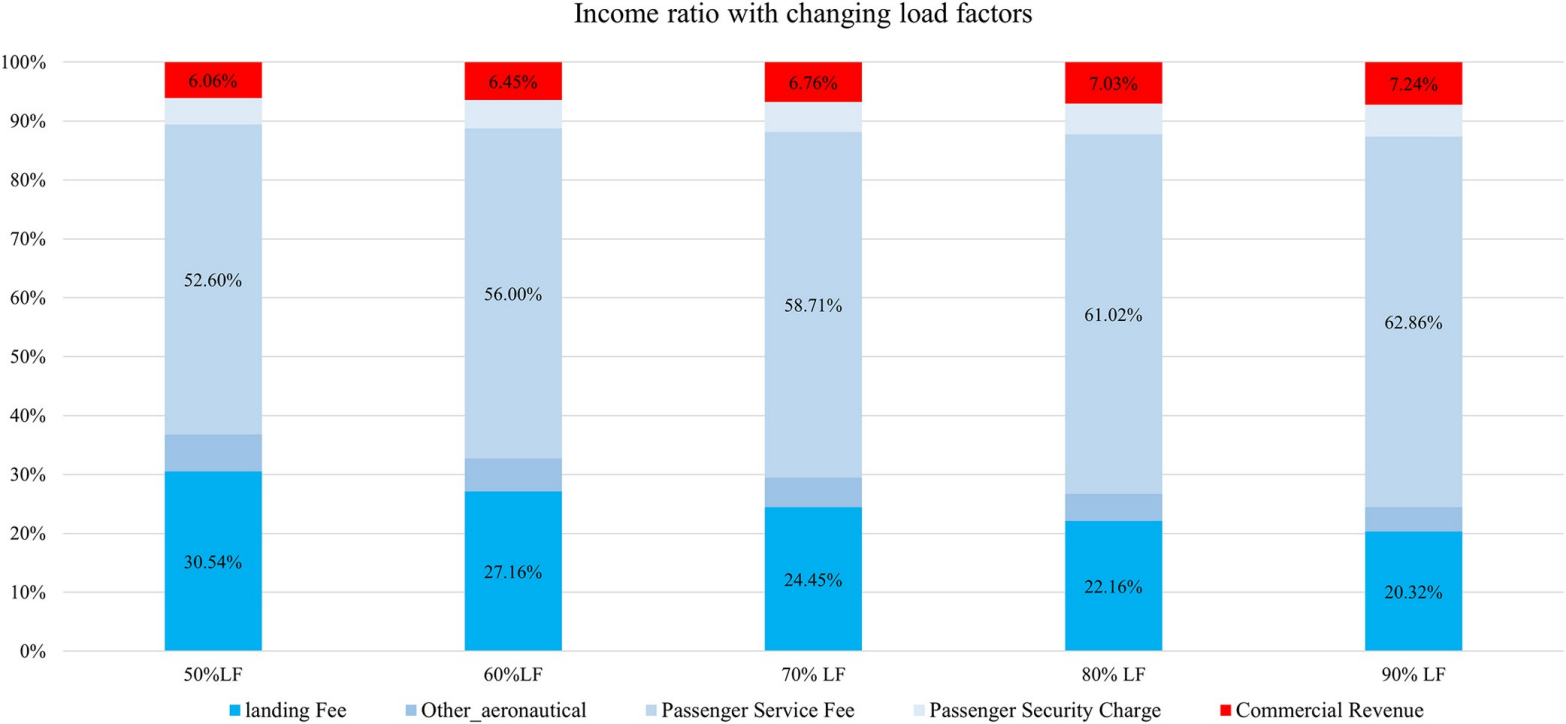


Using the route-level planning diagram, we have calculated revenue of each income source from aeronautical and commercial activities per movement without discount; the ratio of each income has been depicted in Fig 8. It can be viewed that aeronautical service charging (in blue) is the dominant income resource for HGH. Although passenger load increased from 50% to 90% in each scenario, passenger-related incomes, such as passenger service fee income and passenger security charges income, and CR grow along with the increased load factors; CR accounts for less than 10%. This is largely because the small value of CR per passengers—$2.38—has limited revenue contribution compared with average PSF and security charges. Further, aircraft-based income—landing fees—cannot be influenced by load factors; thus, the value of landing fees is a constant during the calculation. However, owing to the growth in passenger-based incomes, the ratio of landing fees consequently dropped from 30% to 20% in Fig 8.

The assumption of average CR might be unreasonable and limited because its actual value for international fights in the airport might be larger than the average level assumed here, but those data are not publicly available. The published data from Zhejiang Tourism indicated that average travel expenditure for inbound tourists was $683.64 in 2017, which is much larger than the average CR. Therefore, the introduction of international flights could make huge contributions to the local economy, but not direct commercial incomes for airports in the short term. Therefore, the objective, as well as the evaluation of the effectiveness of the incentive program, should be considered when airports consider incentive programs. Nevertheless, landing fees and passengers service fees contributed the most to revenues and are the two most prominent cost factors for airline ticket pricing. Therefore, there are two options for HGH to entice airlines’ participation in RD: (1) landing and taking-off fee-based discounts (LF) and (2) passenger service fee-based discounts (PSF).

**Fig 9:**
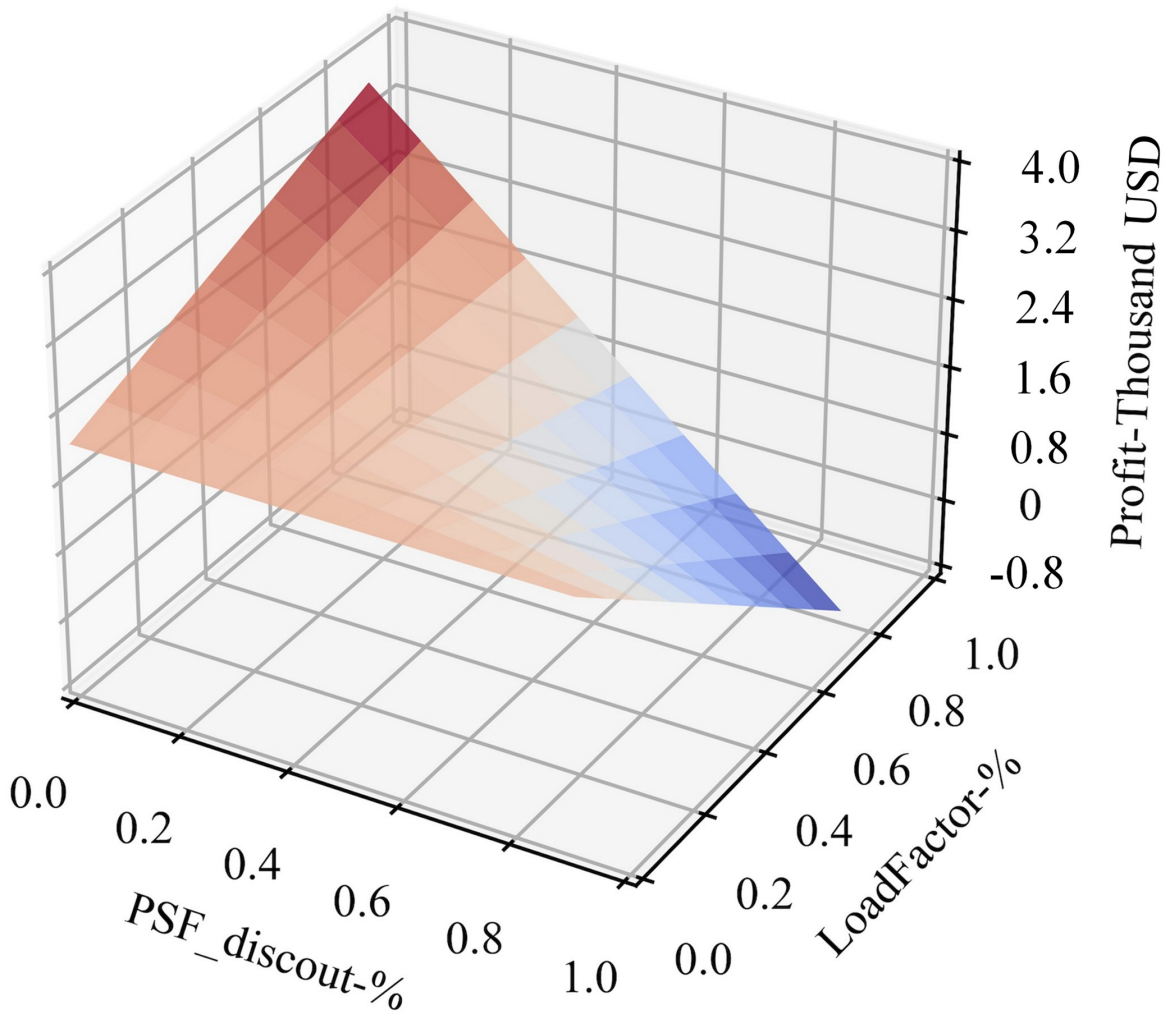


Using the assumptions and relationships in the DPM model above, we have depicted the relationship of profit with changing discounts and load factors per movement under landing fee and passenger service fee plans, as shown in Fig 9. It is expected that when airports waive charging fees and have the best load factors for each movement, airports earn the most money. The more discounts passed to airlines, the less profit made per movement for the airport, although HGH still makes profits under the RD program, mostly because of increasing passenger loads. However, HGH loses money when giving high percentage discounts for PSF. The more passengers carried under the RD program, the less discount can be passed onto airlines if the airport expects to sustain profitability because PSF accounts for more than 50% of income per movement (as shown in Fig 8). It has remarkably positive relations with passenger loads—10% increased load factor resulted in almost 3% growth in passenger service fee income. In the following sections, we will compare these two plans under the incentive settings above.

#### Structure scenarios and results analysis

From the perspective of airport investment planning, managers and practitioners should consider various uncertainties of the new routes. Load factor is among the most important because it directly reflects market potential, which will determine whether new routes are financially sustainable after the termination of incentives. It also influences the effectiveness and performance of airport. Therefore, we developed optimistic and pessimistic scenarios for the incentive planning options landing fee waiver and PSF discount. For the pessimistic scenarios, load factor will decrease10% every year, while for the optimistic case, load factor will increase 10% every year. We assume that the airport will provide a 100% fee discount in the first year, 60% in the second year, and 20% in the third year. So, the standard scenario is that the load factor maintains the industrial standard level (70%) for the whole incentive duration. Table 4 shows the calculation results for the two plans considering the changing of discount and load factor under the optimistic, standard, and pessimistic conditions.

**Table 4:** Results for the standard, optimistic, and pessimistic scenarios.

Incentive options	Scenarios	Year	Disc.	SLF	RD cost (USD)	CR (USD)	Profit (USD)	Local revenue (USD)	Pax. Traffic	RD cost/Pax (USD)	CR /RD costs	Profit /RD cost
**LF Waiver**	**Optimistic**	Y1	100%	70%	2,860.04	1,509.85	6,644.66	432,058.56	632.00	4.53	0.52	2.32
		Y2	60%	80%								
		Y3	20%	90%								
	**Standard**	Y1	100%	70%	2,860.04	1,318.73	6,169.17	377,367.61	552.00	5.18	0.46	2.16
		Y2	60%	70%								
		Y3	20%	70%								
	**Pessimistic**	Y1	100%	70%	2,860.04	1,132.38	5,705.56	324,043.91	474.00	6.03	0.39	1.99
		Y2	60%	60%								
		Y3	20%	50%								
**PSF Discount**	**Optimistic**	Y1	100%	70%	7,423.21	1,509.85	2,081.49	432,058.56	632.00	11.75	0.20	0.28
		Y2	60%	80%								
		Y3	20%	90%								
	**Standard**	Y1	100%	70%	6,867.50	1,318.73	2,161.71	377,367.61	552.00	12.44	0.19	0.31
		Y2	60%	70%								
		Y3	20%	70%								
	**Pessimistic**	Y1	100%	70%	6,328.39	1,132.38	2,237.21	324,043.91	474.00	13.34	0.18	0.35
		Y2	60%	60%								
		Y3	20%	50%								

As we can observe from Table 4, passenger load positively related items, like total CR and total revenue, increased from pessimistic to optimistic scenarios along with passenger traffic for the two planning options. The negative relation with passenger loads resulted in the average value of RD cost per passenger decreasing from pessimistic to optimistic scenarios ($5.18 to $4.53), when the landing fee waiver plan remains the same in different scenarios. However, it decreased from$13.34 to $11.75 under PSF option, although the RD cost increased under the PSF option ($6328.39 to $7423.21). This result indicates that the higher increase rate of passengers enables the airport to enjoy a lower average investment cost per passenger. The greater the passenger load, the more RD cost meant to the airport, and the less total profit was made under PSF. This is largely owing to the structure of airport cost–revenue that the discounted fees from PSF could not be compensated by other income sources, such as CRs from passenger spending in the airport. For the landing fee waiver, because investment related to airframe remained the same, that total profit is higher for the optimistic scenarios than for the pessimistic case, along with passenger load growth. Thus, the value of investment return indicated by the ratio of profit and investment is also larger in optimistic scenarios. This value has the opposite trend under PSF; pessimistic has a better result owing to the larger investment in the optimistic scenario.

We can observe that the ratio of CR to RD cost in both plans are less than 1, which indicates that the money spent on incentivizing airlines could not be compensated by CR generated by passengers because of the small average CR ($2.38). Hence, it is likely that cross-subsidy between aeronautical and non-aeronautical revenue is not satisfying, as expected for HGH in this research This result raises consistency concerns for previous studies; collusion between airports and airlines might fail, because some airports might not be capable of raising commercial businesses and incentivized passengers might not be willing to spending more in the airport [27, 28]. Nevertheless, from the perspective of airport management and planning, HGH should explore more strategies and construction to develop the commercial revenue sources, which is still less regulated in the current environment because there is no single-till or dual-till limitation for airports under the pricing regulation within the Chinese aviation industry.

**Fig 10:**
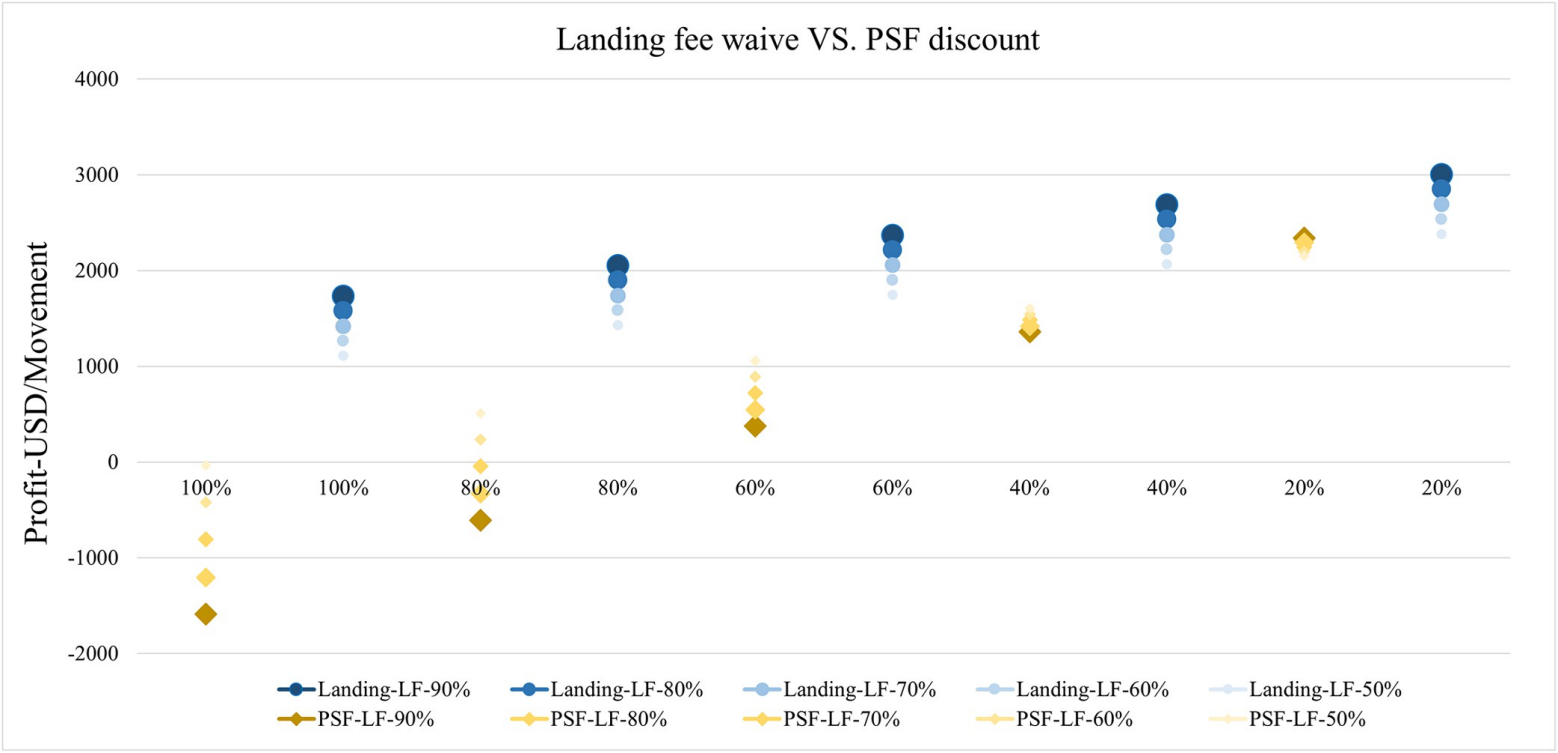


Therefore, when HGH is considering RD strategies to generate profit, a landing fee waiver is suggested because the ratio of profit to RD cost is significantly higher for landing fee waiver plan, as shown in Table 4. Moreover, Fig 10, which illustrates and compares the trend of profits with varying load factors and discounts for those two incentive options, indicates the dominant advantage of landing fee waiver incentive mechanism in profit per movement. This result also explains why landing fee waivers have been the most common instrument for airports to negotiate with airlines. Although the purpose of RD is to increase new services via making airport ‘attractive’ to airlines with charging fee discount, PSF discounts cost the airport more, but offer greater cost-savings for airlines. Hence, if airlines pass the discount benefits to passengers with lower ticket prices to stimulate and sustain route demands, this will generate more income for the airport through higher stimulated demand and higher passenger loads. The primary concern is that passing on rate cuts to passengers is out of airports’ control and is the strategy decision of airlines. Therefore, airports should balance the trade-off between program profit and airlines’ willingness to serve and pass on rates, and then make the detailed rules and eligibilities for RD programs.

## Conclusion

The challenges posed to airports today require the use and design of more multifaceted performance measurement/management systems that can trigger decision-makers’ learning and coordination, strengthen their aptitude in framing dynamic complexity, and support them in pursuing sustainable outcomes. An airport is a complicated system that involves many stakeholders, such as various customers (FSC and LCC) and different users (international passengers/domestic passengers). Hence, a DPM-based approach has been applied to help decision-makers conduct strategy planning and performance measurement. The DPM models built using the RD value framework and the DPM theory have visualized the complexity of route development, the interactions between performance drivers, and the deployment of resources, as well as uncertainties and limitations for the success of RD, such as airlines’ willingness to serve, the duration of the service, passengers’ willingness to pay in the airport, and legal or regulatory conditions, such as incentive regulation and airline entry restriction.

The DPM model built in our paper has shown the important dynamic processes and insights in an empirical study. Therefore, these models could be considered instruments supporting strategies planning and decision-making to frame and solve airport attractiveness promotion problems via RD. Further, the models developed here also provide a framework and indicators to evaluate the RD programs’ effectiveness, which is a challenge owing to the specific programs or organizers. Meanwhile, our work stressed the same concerns as past studies on the benefit of airports’ incentive to airlines—whether incentives could be considered a success in internalizing a positive demand externality between aeronautical and non-aeronautical services [52]. This finding should be treated with caution because it uses a route-level calculation. As the impact of route investment on the enhancement of capacity utilization, the growth of passenger and flight volume, and the increase of commercial income should be estimated on either the program or the airport level.

Nevertheless, the individual cost–revenue estimation provides the general method and insights for HGH to conduct similar scenarios analysis and to collect them for RD investment budget and incentive planning. Hence, HGH should have a deeper understanding of the available routes and the potential airlines with which to cooperate. Particularly, HGH could intergrade the market strategy of its local carrier—Zhejiang Loong Airline—which is based in HGH and has strong support from the Zhejiang Province to develop air travel. Concurrently, Loong Airlines is devoting efforts to explore more international markets because it has fewer international routes compared with other airlines based in HGH. Combined with the CAAC’s preferential policies for new carriers when applying international new routes, cooperation with Loong Airline will enable HGH to expand international services much more simply.

This work clearly has some limitations; qualified generic modeling cannot be used directly to address specific problems for prescribed airports, which are distinguished in terms of characteristics involving facilities, locations, ownership and management, financial structures, and operating environments. Another shortcoming is that we only explored the trade-off between incentives and airport revenues on the route level owing to data accessibility issues. However, this paper is the first step toward enhancing the complicate process modeling of RD from a system perspective.

For future work, the cause–effect relation on RD should be identified and quantified for the system simulation and calibration over a longer period. The program planning could be conducted along with the predicted impacts of RD on passenger and flight traffic, capacity, revenue, and local economy. Additionally, indicator systems to evaluate the effect of RD is suggested and could be built using the model developed in this work. Those indicators could be used for those airports that have launched programs to estimate, diagnose, and revise their incentive mechanisms. However, quantitative studies require more data accessibility, which is particularly an issue because airport charging and airline strategy decisions are not transparent and financial data are not always available from the authorities.

## Supporting information

S1 File (DOCX)

S1 Data (XLSX)
