# Supplementary material for: Airport route development strategy planning and performance measurement with a dynamic performance management framework
Source: PLoS One. 2022 Jul 12;17(7):e0271452. doi: 10.1371/journal.pone.0271452 (PMC9275706; doi:10.1371/journal.pone.0271452)
Supplement: S1 File — (DOCX) [file pone.0271452.s001.docx]

S1: HGH bond credit rating report.

This report is available: <http://www.lhratings.com/reports/B007954-P43394-2019-GZ2020.pdf>.

S2: Airport charging document published by CAAC.

This regulation published by CAAC is available: <https://www.ccaonline.cn/zhengfu/gf-zhengfu/389722.html>.

**Table S2. Charging items and rules in HGH for flights operated by foreign airlines^！！！^.**

| **Charge Rules**  **Charge Items** | **Charges** | | | | |
| --- | --- | --- | --- | --- | --- |
| 1. **Weight based (T)** | MTWO (T) | | | | |
|  | Up to 25 | 26–50 | 51–100 | 101–200 | Over 200 |
| **LTC**  **(USD)** | 296.22 | 325.84 | 325.84 +5.92*(T–50) | 622.06+ 6.52*(T–100) | 1273.73 + 8.29*(T–200) |
| 1. **Time-based (t)** | | | | | |
| **PC(USD)** | Up to 2 hours | | | -- | |
|  | More than 2 hours | | | 15% LTC | |
| **BC(USD)** | Up to 1 hour | | | $29.62 | |
|  | More than 1 hour | | | 29.62 + 14.81 * (t–1) / 0.5 | |
| **PSF(USD)** | $10.37 per passenger | | | | |
| **PSC(USD)** | $1.78 per passenger | | | | |

**^！！！^**Foreign airlines refer to airlines registered out of mainland China.

S3: Route resource regulation published by CAAC.

This document is available on: <http://www.caac.gov.cn>.

S4: Narita international airport incentive program. This route development program, please check: <https://www.naa.jp/en/2017/03/31/20170323-incentive_en.pdf>.

S5: International route subsidies launched by officials in China.

This information is available: <https://www.jiemian.com/article/1864197.html>.

S6: Figure 7 has been drawn by using the USGS National Map Viewer (<http://viewer.nationalmap.gov/viewer/>) as the basemaps. The Figure 7 is a screenshot under the street mode of the basemaps. Measuring tool is used to show the 200km and 300km serving circle. Figure 7 is just the diagram to show the location of airports surrounding Hangzhou Xiaoshan International Airport within the. Yangtze River Delta Region.
